# Iron in Micronutrient Powder Promotes an Unfavorable Gut Microbiota in Kenyan Infants

**DOI:** 10.3390/nu9070776

**Published:** 2017-07-19

**Authors:** Minghua Tang, Daniel N. Frank, Audrey E. Hendricks, Diana Ir, Fabian Esamai, Edward Liechty, K. Michael Hambidge, Nancy F. Krebs

**Affiliations:** 1 Section of Nutrition, Department of Pediatrics, University of Colorado School of Medicine, Aurora, CO 80045, USA; Michael.Hambidge@ucdenver.edu (K.M.H.); Nancy.Krebs@ucdenver.edu (N.F.K.); 2 Division of Infectious Disease, University of Colorado School of Medicine, Aurora, CO 80045, USA; Daniel.Frank@ucdenver.edu (D.N.F.); Diana.Ir@ucdenver.edu (D.I.); 3 Department of Mathematical and Statistical Sciences, University of Colorado Denver, Denver, CO 80204, USA; Audrey.Hendricks@ucdenver.edu; 4 Department of Biostatistics and Bioinformatics, Colorado School of Public Health, University of Colorado Denver, Aurora, CO 80045, USA; 5 School of Medicine, Moi University, P.O. Box 4606, Eldoret 30100, Kenya; fesamai2007@gmail.com; 6 School of Medicine, Indiana University, 705 Riley Hospital Drive, Room 5900, Indianapolis, IN 46202, USA; eliecht@iu.edu

**Keywords:** infants, iron, microbiome, multiple micronutrient powder

## Abstract

Iron supplementation may have adverse health effects in infants, probably through manipulation of the gut microbiome. Previous research in low-resource settings have focused primarily on anemic infants. This was a double blind, randomized, controlled trial of home fortification comparing multiple micronutrient powder (MNP) with and without iron. Six-month-old, non- or mildly anemic, predominantly-breastfed Kenyan infants in a rural malaria-endemic area were randomized to consume: (1) MNP containing 12.5 mg iron (MNP+Fe, *n* = 13); (2) MNP containing no iron (MNP−Fe, *n* = 13); or (3) Placebo (CONTROL, *n* = 7), from 6–9 months of age. Fecal microbiota were profiled by high-throughput bacterial 16S rRNA gene sequencing. Markers of inflammation in serum and stool samples were also measured. At baseline, the most abundant phylum was Proteobacteria (37.6% of rRNA sequences). The proteobacterial genus *Escherichia* was the most abundant genus across all phyla (30.1% of sequences). At the end of the intervention, the relative abundance of *Escherichia* significantly decreased in MNP−Fe (−16.05 ± 6.9%, *p* = 0.05) and CONTROL (−19.75 ± 4.5%, *p* = 0.01), but not in the MNP+Fe group (−6.23 ± 9%, *p* = 0.41). The second most abundant genus at baseline was *Bifidobacterium* (17.3%), the relative abundance of which significantly decreased in MNP+Fe (−6.38 ± 2.5%, *p* = 0.02) and CONTROL (−8.05 ± 1.46%, *p* = 0.01), but not in MNP-Fe (−4.27 ± 5%, *p* = 0.4445). *Clostridium* increased in MNP-Fe only (1.9 ± 0.5%, *p* = 0.02). No significant differences were observed in inflammation markers, except for IL-8, which decreased in CONTROL. MNP fortification over three months in non- or mildly anemic Kenyan infants can potentially alter the gut microbiome. Consistent with previous research, addition of iron to the MNP may adversely affect the colonization of potential beneficial microbes and attenuate the decrease of potential pathogens.

## 1. Introduction

Iron deficiency (ID) and iron deficiency anemia (IDA) are among the most prevalent micronutrient deficiencies in the world [1]. Iron fortification in the form of oral micronutrient powders (MNP) is a common corrective strategy to prevent ID, IDA, or other micronutrient-related deficiencies, especially in low-resource settings [1,2]. However, iron supplementation has been associated with increased occurrence of adverse events, such as infectious diseases and inflammation, which may lead to increased mortality, especially in malaria-endemic areas. For example, the trial conducted in Pemba, Zanzibar [3] found that iron supplementation (12.5 mg/day) was associated with more death and hospitalization in infants and young children compared with the placebo group. Moreover, a recent trial in Pakistan also showed that MNP+Fe resulted in an increased proportion of days with diarrhea and increased incidence of bloody diarrhea in older infants and toddlers [4]. 

Although iron is an essential micronutrient for infant growth and neurodevelopment, it is also an indispensable nutrient for many pathogenic bacteria in the gut, including *Salmonella* spp., *Shigella* spp., and pathogenic *E. coli* [5]. Unabsorbed iron in the gut, usually caused by excessive iron intake (by fortification or supplementation), fosters growth of these pathogenic strains [6] and could potentially modify the balance of microbial species inhabiting the gut, and may thereby pose long-term health consequences. However, research assessing the gut microbiome in relation to iron supplementation in infants is quite limited. An earlier study [7] conducted in young infants (0–3 months) found that infants consuming iron-fortified cow’s milk (5 mg/L) had lower counts of bifidobacteria and higher counts of *Escherichia coli*, compared with infants consuming non-fortified cow’s milk or breastmilk. Bifidobacteria are common probiotics and strongly sequester iron. Certain high iron-binding strains of bifidobacteria inhibit the growth of iron-dependent pathogens, including *Salmonella typhimurium* (*S. typhi*) and *Escherichia coli* O157:H45 [8]. Our research group conducted a controlled feeding study in older U.S. breastfed infants and found a greater decrease of the genus *Bifidobacterium* over time in infants consuming iron-fortified cereal from 5–9 months of age [9]. This alteration of the gut microbiota in favor of less beneficial and more potentially pathogenic strains, could directly affect the host’s health status. Indeed, a six-month, double-blind, randomized, controlled trial in Cote d’Ivoire reported that iron fortification (20 mg/day) not only led to an unfavorable gut microbiome profile, but also increased gut inflammation in anemic African children (6–14 years old) [10]. Furthermore, a recent trial by Jaeggi et al. in primarily anemic Kenyan infants [11] receiving 12.5 or 2.5 mg/day of iron in the form of MNP found that iron supplementation increased *Escherichia/Shigella* and *Clostridium* accompanied by elevated intestinal inflammation status. These studies, although limited and heterogeneous in design, location and age group, suggest the urgent need to further evaluate the effects of iron supplementation on the gut microbiome, especially during infancy when early microbiota colonization is taking place and in non-anemic infants who may not need iron supplementation and are, thus, more susceptible to the potential adverse effects of iron. 

The main objective of this study was to assess the effects of iron fortification in the form of MNP on the gut microbiome and inflammation status in six-month-old, non- or mildly-anemic Kenyan infants. Our primary hypothesis was that iron supplementation would increase the abundance of pathogenic bacteria and decrease the abundance of beneficial bacteria over a three-month intervention. Systemic and intestinal inflammation markers were hypothesized to also increase over time. 

## 2. Materials and Methods 

### 2.1. Study Design

This study was conducted in rural Kenya, approximately 150 km from Eldoret. Among the villages where subjects were recruited, the majority of the population lived below the World Bank-defined poverty level, and the prevalence of malaria is considered to be endemic. Details of the environment were published previously [2]. Study subjects were recruited from the birth registry of the Global Network for Women’s and Children’s Health [12]. The registry administrators provided a list of 45 consecutive infants at this age from their registers based on the study inclusion criteria (see below). For the purpose of identifying participants, the study field staff and the registry administrator visited each infant’s home before enrollment [2].

This was a double-blind, individually-randomized, controlled trial with two primary aims: (1) To identify the effects of iron on the gut microbiome and inflammation status of the host in non- or mildly-anemic infants; and (2) To determine the effects of iron in MNP on zinc absorption and the size of the exchangeable zinc pool, as an indicator of zinc status, after three months of home fortification with daily MNP in maize-based diets [2]. In brief, subjects (15 per group) were randomized to one of the three MNP groups: (1) The MNP+Fe group: MNP with iron (12.5 mg/day) and zinc (5 mg/day); (2) The MNP-Fe group: the same MNP, except no iron; (3) the CONTROL group: placebo with no micronutrients. The intervention started at 6 months of age and ended at nine months of age. This study was approved by the Colorado Multiple Institutional Review Board and Moi University Institutional Review Board. Potential families were visited and educated on the proposed study before the enrollment visit and the study nurse or lab technologist obtained written informed consent from the parents/guardians plus witness upon confirmation that the child met the inclusion and exclusion criteria. Forty-five pieces of paper with letters marked A, B, or C to represent the three study arms were rolled into balls and placed in a plastic container. The pieces were mixed thoroughly through shaking of the container. An independent person then picked the pieces one by one from the container. The unique number-letter became the subject and group assignment for each individual [2]. The study was registered in ClinicalTrials.gov NCT02101723. Findings here are from subjects (*n* = 33 total) who had fecal samples collected at both time points. 

### 2.2. Subjects

Enrollment of each subject was prior to six months of age. Inclusion criteria: (1) born at term with birth weight >2500 g; (2) no apparent congenital anomalies; (3) immunization up to date; (4) normal family social situation such that they were able to comply with study requirements; (5) agree to weekly visits for MNP distribution and data collection, as well as stool, urine, and blood collections; (6) hemoglobin (Hb) ≥ 10 g/dL; (7) breastfeeding at enrollment and intent to continue during the study period; and (8) negative for malaria parasites based on blood smear. Exclusion criteria: (1) acute malnutrition, with weight for length Z score (Z: number of standard deviations away from the mean) ≤ 2 SD by the World Health Organization (WHO); (2) current or planned use of infant formula or other nutrient fortified products; (3) current or planned use of iron or zinc supplements; (4) moderate or severe anemia (Hb < 10 g/dL); (4) previous hospitalization for malaria within the last four weeks; (5) persistent diarrhea (per WHO, >3 loose stools/day lasting ≥14 days) [2]. There was no use of antibiotics reported at any time points. 

### 2.3. Micronutrient Powder and Diet

Micronutrient composition of the two intervention MNPs and the placebo are listed in Table 1. Iron was in the form of ferrous fumarate and was microencapsulated. The placebo was made of inactive powder. Laboratory analysis at the University of Colorado (UC) verified the accuracy of the zinc and iron contents of the products. All of the MNP sachets were de-identified by the manufacturer (Hexagon Nutrition Pvt. Ltd., Mumbai, India) to both the study subjects and the study field staff. During the intervention, seven MNP sachets were provided at weekly home visits to the mother for the following week, plus three extra in case of a delay in the next visit. Mothers received instructions to give one sachet each day and to add the entire contents of the sachet to one meal. Infants’ diets were directly observed over the course of the trial as part of the zinc absorption study (aim 1 of this trial). The foods were primarily a maize-based porridge, ugali (maize), and vegetables [2]. During the weekly home visits, the subjects were followed up by the nurse or clinical officer to check the general health status, ensure compliance, and answer questions that mothers had [2]. To monitor compliance, the nurse or clinical officer collected empty sachet packets and those that were not consumed (if any).

### 2.4. Sample Collections

For the purpose of this analysis, fecal samples were collected at baseline (six months) and the end of the intervention (nine months). Research personnel from UC trained the local field researchers in the fecal collection procedures. Each stool sample was collected from cloth diapers fitted with biodegradable liners, which effectively collected stool, but allowed the passage of urine [9]; two duplicate stool samples were placed in separate sterile collection tubes (Sarstedt, Newton, MA, USA) containing RNA*later* (ThermoFisher Scientific, Waltham, MA, USA). The field workers assisted mothers with stool collections, using clinical grade gloves and sterile fecal swabs to avoid microbial contamination. A study nurse at baseline (six months) and the end of the intervention (nine months) also collected blood samples using heparin vacutainers and butterfly needles. Blood tubes were incubated without shaking at room temperature for 45 min before being centrifuged for serum separation. Both fecal and blood samples were placed on ice at the home visits, and were held in a −20 °C freezer for overnight stay at the local hospital until transport the next day to the −70 °C freezer at Moi University (Eldoret, Kenya), using an ice-packed vaccine carrier. Specimens were shipped on dry ice to the University of Colorado.

### 2.5. Anthropometric Measurement

Anthropometric measurements, including weight and length, were measured at baseline (six months) and the end of the intervention (ninr months) in triplicate by the research staff. Details of the weight and length measurements were published elsewhere [2].

### 2.6. Fecal Sample Analysis

#### 2.6.1. 16S Amplicon Library Construction

Microbiome sequencing was conducted at the Microbiome Research Consortium at University of Colorado Denver. Total community genomic DNA was prepared from ~50 mg of stool using the UltraClean Fecal DNA Isolation Kit (MO BIO Laboratories, Carlsbad, CA, USA). Bacterial profiles were determined by broad-range amplification and sequence analysis of 16S rRNA genes following our previously-described methods [13,14]. In brief, amplicons were generated using primers that target approximately ~270 base pairs of the V4 variable region of the 16S rRNA gene (primers 534F: GTGCCAGCMGCCGCGGTAA and 805R: GACTACCAGGGTATCTAAT) [15,16]. Polymerase chain reaction (PCR) products were normalized using a SequalPrep^TM^ kit (Invitrogen, Carlsbad, CA, USA), pooled, lyophilized, purified and concentrated using a DNA Clean and Concentrator Kit (Zymo, Irvine, CA, USA)*.* Pooled amplicons was quantified using Qubit Fluorometer 2.0 (Invitrogen, Carlsbad, CA, USA). The pool was diluted to 4 nM and denatured with 0.2 N NaOH at room temperature. The denatured DNA was diluted to 15 pM and spiked with 25% of the PhiX (Illumina, Inc., San Diego, CA, USA) control DNA prior to loading the sequencer. Illumina paired-end sequencing was performed on the Illumina Miseq platform (Illumina, Inc., San Diego, CA, USA) with versions v2.4 of the MiSeq Control Software, using a 500 cycle version 2 reagent kit (Illumina, Inc., San Diego, CA, USA).

#### 2.6.2. 16S rRNA Sequence Analysis

Illumina MiSeq paired-end reads were aligned to human reference genome hg19 with bowtie2 and matching sequences discarded [17,18]. As previously described, the remaining non-human paired-end sequences were sorted by sample via barcodes in the paired reads with a python script [14]. The sorted paired reads were assembled using phrap [19,20]. Pairs that did not assemble were discarded. Assembled sequence ends were trimmed over a moving window of five nucleotides until the average quality met or exceeded 20. Trimmed sequences with more than one ambiguity, or shorter than 350 nt, were discarded. Potential chimeras identified with Uchime (usearch6.0.203_i86linux32) [21] using the Schloss [22] Silva reference sequences were removed from subsequent analyses. Assembled sequences were aligned and classified with SINA (1.3.0-r23838) [23] using the 418,497 bacterial sequences in Silva 115NR99 [24] as reference configured to yield the Silva taxonomy. Operational taxonomic units (OTUs) were produced by clustering sequences with identical taxonomic assignments. This process generated 12,309,588 sequences for 150 samples (median: 82,064 sequences/sample; minimum sample size: 3619; maximum sample size: 168,818) exclusive of negative controls which were near zero. The median Goods coverage score was ≥99% at the rarefaction point of 15,000. The software package Explicet (v2.10.5, www.explicet.org) [25] was used for data management, display, and analysis (rarefied values for median Good’s coverage). 

#### 2.6.3. Fecal Calprotectin Analysis

A quantitative PCR (QPCR) gene expression assay was used to quantify expression of the calprotectin gene, a marker used for detection of inflammation in the intestines. Calprotectin protein levels were not measurable because samples were stored in RNA*later*. All reactions were performed in 96-well plates using the BIORAD CFX96 instrument. PCR reactions contains 10 μL DyNAmo ColorFlash probe QPCR mastermix (ThermoFisher Scientific, Waltham, MA USA), 7 μL sterile nuclease-free water (ThermoFisher Scientific, Waltham, MA, USA), 1 μL of the S100A8 primers, and 2 μL of sample template. The following primers were used: Glyceraldehyde 3-phosphate dehydrogenase (GAPDH) Hs.PT.51.22847819.gs and S100A8 Hs.PT.51.19654111.gs. Internal normalization was performed using the GAPDH housekeeping gene. Fold expression change of S100A8 relative to GAPDH was calculated using the ∆∆Ct method [26]. 

### 2.7. Blood Sample Analysis

Markers of systemic inflammation, including α 1-acid glycoprotein (AGP), C-reactive protein (CRP), TNF-α, IL-6 and IL-8 were analyzed in serum by R & D Systems Quantikine Enzyme-linked Immunosorbant Assay (ELISA) in the Pediatric Nutrition Laboratory at University of Colorado Denver. Iron status of the subjects was also measured and already reported [2]. 

### 2.8. Statistical Analysis

Sample size calculations were based on the primary hypothesis that iron supplementation would increase the abundance of pathogenic bacteria and decrease the abundance of beneficial bacteria over a three-month intervention. We estimated a sample size of 10–15 per group to detect a 10% difference of relative abundance of a generic organism between groups, considering a SD of 15% with alpha = 0.05. This sample size gave a power of 71–89%. 

Statistical analyses were performed using SAS (version 9.3; SAS Institute Inc., Cary, NC, USA). Group means are presented as mean ± SD. Baseline parameters were compared using one-way Analysis of Variance (ANOVA) and Tukey’s multiple comparisons as the post-hoc analysis when needed. Gender was included as a covariant in the subsequent analysis and results remained unaffected. Repeated measures ANOVA were used to evaluate the main effects of time, group, and their interactions on the dependent variables (i.e., microbiome and inflammation markers). Tukey’s multiple comparisons test was used to compare values between groups as the post hoc analysis (adjusting for multiple groups). A paired *t*-test was used to compare change over time within each group. Relative abundance (%) of the microbiome data at the phyla and genus levels were analyzed. The four most abundant phyla, Actinobacteria, Firmicutes, Bacteroidetes, and Proteobacteria, were analyzed. At the genus level, three genera of interest *Clostidium, Escherichia/Shigella,* and *Bifidobacterium* were analyzed based on previous research by Jaeggi et al., [11] of a comparable design. Multiple comparisons were adjusted based on the number of models tested (three microbiome strains and seven inflammation markers). Thus, an adjusted *p*-value of less than 0.005 is considered significant after Bonferroni correction (0.05/10 tests = 0.005). *p*-values between 0.005 and 0.05 were also reported and considered nominally significant.

## 3. Results

### 3.1. Subject Characteristics

A subgroup of 33 subjects who completed the fecal sample collections at both time points were included in this analysis: MNP+Fe: *n* = 13; MNP-Fe: *n* = 13; CONTROL: *n* = 7. The discrepancy of sample sizes between groups did not appear to be related to the intervention: (1) the intervention was double-blind; (2) some stool samples were missing labels during shipment or the quantity was not adequate. Subjects’ demographics and anthropometric data are summarized in Table 2. In brief, there were no differences in demographic or anthropometric measurements between groups. Both weight and length increased over time as expected among all subjects (data not shown) with no group differences. No cases of malaria were identified in the subjects during the study period. Compliance monitoring for the assigned MNP indicated > 90% consumption among the participants [2]. 

There was no difference between groups for hemoglobin, ferritin, soluble transferrin receptor, plasma zinc, or exchangeable zinc poo (EZP) at baseline. After the intervention, plasma zinc concentration decreased without differences between groups. Serum ferritin did not change over time or between groups, but soluble transferrin receptor demonstrated a significant increase, with trend for greater increase in the control group [2].

### 3.2. Microbiome Changes over Time

The Good’s index of each sequence library at six and nine months for the three groups were all higher than 99%, indicating that most biodiversity was captured in each library (subject), and that the depth of sequencing was sufficient to represent the biodiversity in the specimens. Ecological indices of richness (S_chao1_) increased overtime from 6–9 months (*p* < 0.0001) but no between-group differences were noted. The increase of diversity corresponded to the subjects’ increase in food variety. These numbers are within the similar range we previously reported in breastfed infants in Denver, CO, USA [9].

**Figure 1:**
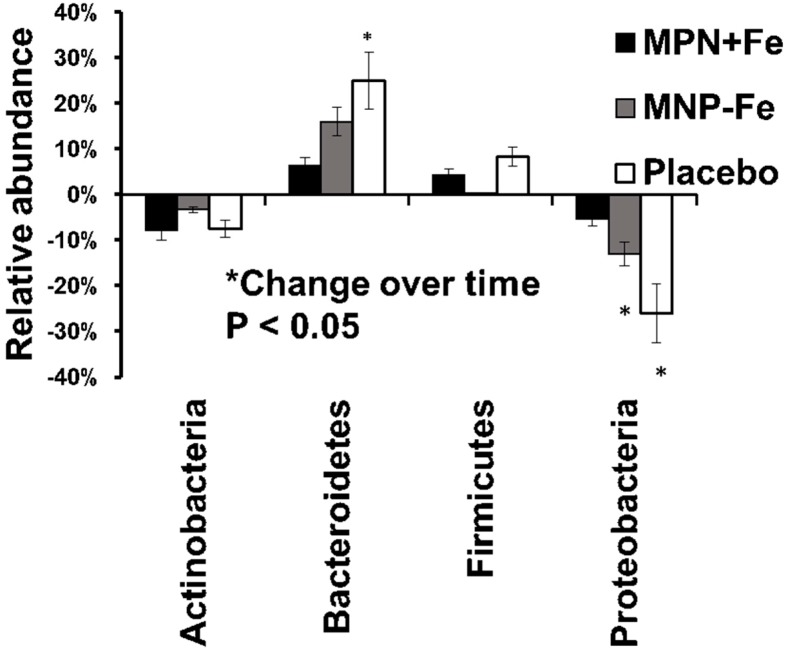
The change of relative abundance at the phyla level.

The four most abundant phyla at baseline were Actinobacteria (20 ± 13% relative abundance [RA]), Bacteroidetes (17 ± 19% RA), Firmicutes (24 ± 13% RA), and Proteobacteria (38 ± 21% RA), wherease the rest of the phyla accounted for less than 1% of 16S rRNA sequences. There was no significant effect of group (*p* = 0.1) at the phylum level but a nominal significant effect of time for Actinobacteria (*p* = 0.02), Bacteroidetes (*p* = 0.009) and Proteobacteria (*p* = 0.008). Figure 1 showed the breakdown of the relative abundance change overtime within each group. When consuming their regular diet plus a placebo MNP over there months (CONTROL), the relative abundance of Bacteroidetes (*p* = 0.04) increased and Proteobacteria (*p* = 0.02) decreased. There was also a borderline decrease of Actinobacteria in CONTROL (*p* = 0.07). The abundance of Actinobacteria also was decreased in MNP+Fe (*p* = 0.02). 

**Figure 2:**
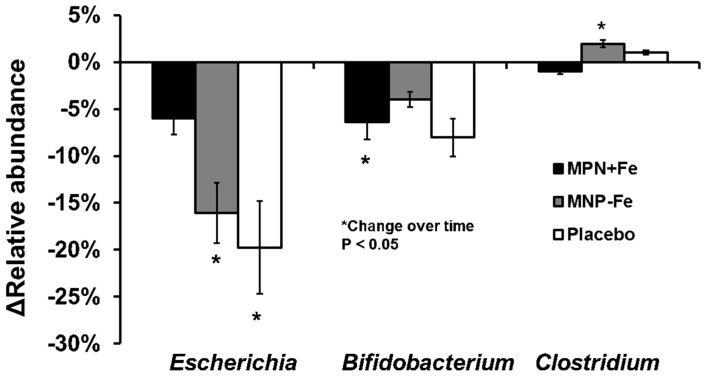
Change of relative abundance at the genus level.

At the genus level at baseline, *Escherichia/Shigella,* (30.1 ± 23%) and *Bifidobacterium* (17.3 ± 11.1%) were the first and second most abundant genera among all groups, while *Clostridium* (1.4 ± 3.5%) was the eighth most abundant genus. As shown in Figure 2, the relative abundance of *Escherichia/Shigella* significantly decreased over time in the MNP-Fe (−16.05 ± 6.9%, *p* = 0.05) and CONTROL (−19.75 ± 4.5%, *p* = 0.01) groups, but not in the MNP+Fe group (−6.0 ± 9%, *p* = 0.41). The relative abundance of *Bifidobacterium* decreased in MNP+Fe (−6.38 ± 2.5%, *p* = 0.02) and CONTROL (−8.05 ± 1.46%, *p* = 0.01), but not in the MNP-Fe group (−4.3 ± 5%, *p* = 0.44). In addition, *Clostridium* increased abundance in MNP-Fe only (1.94 ± 2%, *p* = 0.007). No significant difference was found for the ratios of *Escherichia/Shigella* to *Bifidobacterium* or *Escherichia/Shigella* to *Clostridium* between groups (log of the abundance). 

### 3.3. Inflammation Markers

AGP and CRP concentrations were previously reported [2]. Table 3 summarizes the serum inflammatory markers AGP, CRP, TNF-α, IL-6, IL-8, and fecal calprotectin at six and nine months of age. Briefly, none of the inflammation markers changed over time or between groups, except for IL-8, which decreased in CONTROL (group-by-time interaction *p* = 0.02). CRP was above the upper limit of the normal range for all groups at both time points. 

## 4. Discussion

This study showed that in 6–9 month old breastfed African infants who were generally not anemic, home fortification with iron containing MNP could alter the gut microbiome in favor of potential pathogens, compared with infants who received MNP without iron or no MNP at all. Jaeggi et al. [11] conducted a double-blind, randomized, controlled trial in Kenyan infants of comparable design as our study, but with a larger sample size. In Jaeggi et al.’s study [11], six-month old Kenyan infants, who had high rates of IDA (64%), were randomized to receive MNP with iron (12.5 or 2.5 mg/day) or MNP with no iron for four months. Their results indicated that iron supplementation increased *Escherichia/Shigella* and *Clostridium* accompanied by elevated intestinal inflammation status [11]. The 12.5 mg Fe/day MNP used had a similar composition with our study, except for lower folic acid. However, limited research has shown that folic acid had minimum impact on the gut microbiome [27]. The 2.5 mg Fe/day MNP in Jaeggi et al.’s study [11] also contained 11 other micronutrient and resulted in a slightly different impact on the microbiome, especially for *Escherichia/Shigella*. This differential impact could be due to the different quantity of iron or the other micronutrients in the 2.5 mg Fe/day MNP, but this cannot be distinguished by the design of the study [11]. Another study [10] by the same group showed that in older African children, 20 mg/day iron fortification resulted in a significant increase in the abundance of enterobacteria, a decrease in lactobacilli, and a significant increase in fecal calprotectin. Findings from a complementary feeding intervention of older infants, with a larger sample size and longer intervention, suggested that iron-fortified cereal was associated with higher systemic inflammation and impaired linear growth; the effect on the gut microbiota was not reported [28]. These studies, although differing from our study by the environment, iron dosage, and subjects’ age, all demonstrated the potential pathogen-promoting nature of elevated iron in the human gut in low-resource settings. 

The four predominant phyla observed in this study were comparable to previous studies conducted by our group [9,29] and others [30]. However, we found a higher abundance of Proteobacteria at 38% compared with other studies [11,30] that reported typical abundances less than 10%. Although comparing the results of microbiome studies can be challenging due to methodological differences, these results suggest the possibility that these Kenyan infants at six months of age were colonized by a potentially more pathogenic microbiome than observed in other environments. In addition to the environment, diet also plays an important role in modulating the microbiome. For instance, children from Europe and rural Africa have been reported to display distinctly different microbial communities [30]. The most profound difference was observed in Bacteroidetes, specifically the genus *Prevotella*, which is known for polysaccharide hydrolysis. This finding is consistent with the fiber-rich diet characteristic of many rural African children, compared with children from Europe. In our study, the relative abundance of Bacteroidetes also increased over time, concurrently with the increase of complementary feeding and food variety, particularly the intake of maize-based foods. 

The three genera *Bifidobacterium*, *Escherichia/Shigella*, and *Clostridium* were chosen for analysis based on previous research by Jaeggi et al. [11] of a comparable design. The genus *Bifidobacterium* is usually predominant in breastfed infants and is one of the beneficial “barrier” strains that prevent pathogen colonization and adhesion to epithelial cells of the host [31]. The abundance of *Bifidobacterium* usually declines with age. In our study, the abundance of *Bifidobacterium* at six months was 17.3% on average, which was much lower than what Jaeggi et al. [11] reported at 63%. This discrepancy could be due to the 16S rRNA gene primer bias [32], although the primers used in this study were designed to minimize the bias in amplifying bifidobacteria [33]. Moreover, our findings are in the range of previous research [30,34], including our own studies [9,35]. In our study, *Bifidobacterium* decreased over time in both MNP+Fe and CONTROL, but not MNP-Fe. The decrease in CONTROL is expected because *Bifidobacterium* usually decreases its abundance with age [31]. Moreover, the decrease in MNP+Fe was consistent with previous research by Jaeggi et al. [11] that also showed a decrease in *Bifidobacterium* abundance from 6–10 months of age with the same daily iron supplementation. However, the study by Jaeggi et al. [11] did not include a control group to reflect the natural change of microbial colonization with time. Thus, at least for *Bifidobacterium*, our study suggests that inclusion of 12.5 mg of iron in MNP did not have additive negative effects to the host. Nonetheless, in the MNP-Fe group, *Bifidobacterium* did not significantly decrease over time, suggesting the potential beneficial effect of MNP without iron, on attenuating the natural decline of *Bifidobacterium* in these infants. Our results also indicated that the genus *Escherichia/Shigella* decreased in abundance in MNP-Fe and CONTROL, but not MNP+Fe. This is an interesting finding because, unlike the study by Jaeggi et al. [11], iron supplementation in our study did not promote the growth of *Escherichia/Shigella* per se, but rather prevented its decrease over time, as observed in MNP-Fe and CONTROL. Furthermore, the abundance of the genus *Clostridium* did not change in MNP+Fe and CONTROL, but only MNP-Fe. Overall, our findings suggest that at the genus level, standard iron supplementation (12.5 mg/day) in the form of MNP affected the gut microbiome in a potentially adverse fashion by inhibiting the natural diminution over infancy of *Escherichia/Shigella.* Meanwhile, MNP without iron had mixed effects on the microbiome by inhibiting the decrease of *Bifidobacterium* while promoting the growth of *Clostridium.* Without sub-phyla level determinations, it is impossible to predict the likely impact of the latter. In our earlier study in Denver, high iron intake was associated with lower *Clostridium* group XIVa, which includes clostridial species that are butyrate-producing and immunomodulatory [9]. 

Although our study found that iron supplementation decreased potentially beneficial strains (e.g., *Bifidobacterium* spp.) and attenuated the temporal decrease of pathogenic strains (e.g., *Escherichia* spp.), no statistically significant effect on inflammation status was found. In CONTROL, IL-8 did decrease over time, but MNP+Fe and MNP-Fe did not significantly increase any inflammation markers, including fecal calprotectin, which previous studies found increased with iron supplementation [10,11]. This lack of a significant change could be due, at least partially, to a relatively small sample size. Additionally, these infants already had a high burden of inflammation at baseline, which may diminish the intervention effect. Moreover, due to the nature of sample storage, fecal calprotectin in this study was measured as gene expression, which did not necessarily reflect protein levels. However, unlike previous studies [10,11], we did not find an increase of the pathogenic strains (*Escherichia/Shigella*, *Clostridium*) with iron supplementation, which may also contribute to the unchanged inflammation status in these infants. 

Strengths of our study include targeting non-anemic infants in rural Kenya, a group that may not need iron supplementation and are thus more susceptible to adverse effects of iron supplementation, especially in the setting of a high inflammatory burden and endemic malaria [3]. In addition, we included a control group to demonstrate the natural change of the gut microbiome in these infants. There are also a number of limitations of this trial. First, our sample size was much smaller compared with that of Jaeggi et al. [11]. Our study was initiated in 2011 and power and sample size were calculated based on very limited research available at that time. Data that demonstrate substantial variability have emerged since and our study appears to be underpowered. Nonetheless, our findings contribute to this important research topic because of our targeted population and inclusion of a control group. Another limitation, common to other studies, was the lack of long-term follow up. Although the relative abundance of some potential pathogens remained with iron supplementation, it is not clear how this would affect the health status of the host in the long term, for example, in relation to growth [28]. Lastly, some of infants (in all groups) had already started complementary foods at baseline, but the foods were likely to have been virtually the same as those consumed during the intervention period. By reporting the change over time within participants, we could partially control the individual variances of the gut microbiome. 

Iron deficiency is the most prevalent micronutrient deficiency in the world and finding an adequate and safe dosage of iron to rectify this deficiency has tremendous public health impact [36,37,38]. Ours was the first randomized, controlled trial that examined the effect of standard and widely-used iron supplementation on the gut microbiome in non- or mildly-anemic African infants. The quantity of iron provided in this study was 12.5 mg/day, which is the standard quantity in commonly-used MNP “sprinkles”. This dosage has been shown to be effective in terms of reducing ID and IDA in older African infants and toddlers. Lower iron doses have also been tested, with the aim of reducing its negative health impacts (e.g., inflammation, diarrhea). For example, MNP with a much lower iron content (2.5 mg/day) was tested in several trials in African children, but results were not promising [39,40]. Additionally, Jaeggi et al. [11] found it unclear whether 2.5 mg/day of iron, compared with 12.5 mg/day, had a better safety profile. Future preventive iron interventions could consider the impact of lower doses, effects of absorption enhancers, such as vitamin C, and/or anti-inflammatory agents, such as Vitamin E [29], to the MNP. Ultimately, additional research needs to be done to determine the most effective dosage with minimum side effects to both infant health and to the developing microbiota. Moreover, longer term studies will be required to link the effects of alterations in the microbiota with clinically-relevant outcomes. 

## Figures and Tables

**Table 1:** Micronutrient contents of the MNP ^1^ per sachet.

	MNP+Fe	MNP-Fe	Placebo
Iron	12.5 mg	0	0
Zinc	5 mg	5 mg	0
Vitamin A	300 μg	300 μg	0
Vitamin C	30 mg	30 mg	0
Folic acid	160 μg	160 μg	0

^1^ Multiple micronutrient powder.

**Table 2:** Demographics and anthropometrics at six months of age ^1^.

	MNP+Fe (*n* = 15)	MNP−Fe (*n* = 15)	Control (*n* = 15)
Male	5 (33%)	7 (47%)	10 (67%)
Female	10 (67%)	8 (53%)	5 (33%)
Weight, kg	7.25 ± 0.25	7.55 ± 0.27	7.49 ± 0.22
WAZ ^2^	−0.39 ± 0.25	−0.16 ± 0.31	−0.21 ± 0.25
Length, cm	65.0 ± 0.7	66.5 ± 0.9	64.6 ± 0.8
LAZ ^2^	−0.68 ± 0.27	−0.19 ± 0.40	−1.01 ± 0.39

^1^ Mean ± SEM; no difference between groups of any parameter; ^2^ WAZ: weight-for-age Z score; LAZ: length-for-age Z score.

**Table 3:** Mean (± SEM) inflammation markers at six and nine months of age ^1^.

Biomarkers (Normal Range)	MNP+Fe		MNP-Fe		Control	
	Six Months	Nine Months	Six Months	Nine Months	Six Months	Nine Months
AGP (mg/dL) (50–120)	87 ± 14 (10)	91 ± 19 (10)	81 ± 17 (7)	81 ± 12 (7)	82 ± 15 (9)	80 ± 13 (9)
CRP (mg/L) (<3)	7.7 ± 4.5 (10)	6.2 ± 3.4 (10)	11.5 ± 5.9 (8)	8.3 ± 4.2 (8)	5.3 ± 1.8 (8)	6.2 ± 1.7 (9)
IL-6 (pg/mL) (<39)	7.5 ± 4.0 (12)	5.1 ± 2.4 (15)	8.8 ± 3.0 (14)	3.2 ± 0.9 (14)	5.8 ± 2.4 (10)	5.0 ± 1.6 (11)
IL-8 (pg/mL) ^2^ (<81.8)	39 ± 11 (12)	30 ± 4 (14)	40 ± 20 (14)	32 ± 8 (14)	51 ± 13 (10)	22 ± 3 (11)
TNF-α (pg/mL) (<3.3)	8.7 ± 1.1 (14)	8.6 ± 0.8 (15)	12.2 ± 3.0 (14)	8.3 ± 1.0 (15)	9.1 ± 1.3 (10)	7.3 ± 1.1 (11)
Fecal calprotectin (fold change)	1.6 ± 0.6 (10)	2.1 ± 0.6 (11)	3.4 ± 0.8 (12)	2.5 ± 0.8 (8)	4.3 ± 1.4 (6)	2.3 ± 1.1 (6)

^1^ Mean ± SEM (n); ^2^ Decreased in CONTROL (group-by-time interaction *p* = 0.02).
